# Connecting the free energy principle with quantum cognition

**DOI:** 10.3389/fnbot.2022.910161

**Published:** 2022-09-02

**Authors:** Yukio-Pegio Gunji, Shuji Shinohara, Vasileios Basios

**Affiliations:** ^1^Department of Intermedia Arts and Science, School of Fundamental Science and Technology, Waseda University, Tokyo, Japan; ^2^School of Science and Engineering, Tokyo Denki University, Tokyo, Japan; ^3^Service de Physique des Systèmes Complexes et Mécanique Statistique and Interdisciplinary Center for Nonlinear Phenomena and Complex Systems (C.P.231 CeNoLi-ULB), Université Libre de Bruxelles, Brussels, Belgium

**Keywords:** free energy minimization, quantum cognition, Bayesian inference, rough set, lattice theory

## Abstract

It appears that the free energy minimization principle conflicts with quantum cognition since the former adheres to a restricted view based on experience while the latter allows deviations from such a restricted view. While free energy minimization, which incorporates Bayesian inference, leads to a Boolean lattice of propositions (classical logic), quantum cognition, which seems to be very dissimilar to Bayesian inference, leads to an orthomodular lattice of propositions (quantum logic). Thus, we address this challenging issue to bridge and connect the free energy minimization principle with the theory of quantum cognition. In this work, we introduce “excess Bayesian inference” and show that this excess Bayesian inference entails an underlying orthomodular lattice, while classic Bayesian inference entails a Boolean lattice. Excess Bayesian inference is implemented by extending the key idea of Bayesian inference beyond classic Bayesian inference and its variations. It is constructed by enhancing the idea of active inference and/or embodied intelligence. The appropriate lattice structure of its logic is obtained from a binary relation transformed from a distribution of the joint probabilities of data and hypotheses by employing a rough-set lattice technique in accordance with quantum cognition logic.

## Introduction

Cognitive predictive behaviors that are found in brain function, biological information processing, and cognitive sciences have been recently described and explained using the free energy minimization principle (Friston et al., 2006; Friston and Kiebel, 2009a,b). However, related cognitive phenomena such as sensory illusions (e.g., due to ambiguity such as in the Necker cube), the conjunction fallacy (e.g., “Linda's fallacy”), the order effect in questionnaire responses, context-dependent decision-making, and the “Guppy effect” in complex concept conjunction and disjunction have been recently described and explained by using quantum cognition principles (Khrennikov, 2001, 2010, 2021; Aerts, 2009; Aerts et al., 2012, 2013, 2019; Busemeyer and Bruza, 2012; Haven and Khrennikov, 2013; Asano et al., 2015; Bruza et al., 2015; Dzhafarov et al., 2016; Ishwarya and Kumar, 2020a,b). Moreover, recent developments in quantum measurement theory provide a general mathematical framework that can accommodate the question order effect and the response replicability effect as well as their combinations. Thus, the generalization of the Wang–Busemeyer quantum-cognition postulates for quantum-like modeling of decision-making and psychology is achieved. An up-to-date discussion of these recent developments and an introduction to this “theory of quantum instruments” can be found in Ozawa and Khrennikov (2021).

It has come to our attention through a reviewer's suggestion that in a recent publication (Zhang, 2021a,b), a newly proposed analytical quantum computing paradigm, called “quantum intelligence” (QI), aims at elucidating the notion of causality concerning the underlying logic of the phenomena under scrutiny.

Predictive algorithms and coding that deal with large problems, i.e., problems that require too much computation to obtain an optimal solution, meet the seriously challenging problem of reducing the search area for the solutions to make their implementation manageable or even possible. Bayesian inference is one of the most powerful techniques that can solve this problem (Arecchi, 2003, 2011). Moreover, it is well known that Bayesian inference can be accurately formulated as an instance, or a case of the free energy minimization principle (Friston et al., 2006; Friston, 2010). The efficacy of Bayesian inference is because it only focuses on an a priori probability distribution assumed by the already given or realized events. These events have been “experienced,” “realized,” or “recorded,” as given, but there is no requirement that *all* events from such an a priori distribution have been “realized.” The relevant events could all, in principle, be hypothetical. In the context of the free energy principle, the prior distribution is implemented by a generative model of the action of the environment on a Markov blanket. As this action is generated outside the Markov blanket, by the dynamics of the environment, it is unobservable “in principle,” i.e., under this prior. Therefore, such actions are duly ignored; obviously, this inherent aspect of Bayesian inference helps to reduce the search space. This is the essence of Bayesian inference.

Furthermore, an embodied mind and/or intelligence (Varela et al., 1991; Varela, 1997) can augment and complement the process of Bayesian inference in biological information processing and even overcome some of its drawbacks (Seth and Friston, 2016; Allen and Friston, 2018; Seth and Tsakiris, 2018; Yon et al., 2019; Walsh et al., 2020). For example, let us assume that you are familiar only with the front face of your preferred singer, say through some photos. Now imagine that you encounter that singer on a street in a town; then, you would naturally be impelled to move your body to try to see the front face. Since you have experienced more front-face photos than photos from other angles, Bayesian inference would ignore the face data that have been experienced less often, and this would make the relation between the singer in the street and the familiar front-face image retrieved by memory stronger; eventually, this would result in the recognition that this person is indeed your preferred singer, provided that the match is close enough. This is a result of the fact that you cannot identify the face seen from an angle (because of uncertainty, missing data, or the frame problem), which implies that there is a disadvantage to Bayesian inference. Moving your body to see the front face of the singer implies that action-motion control and determining the correct placement of the body can address this disadvantage of Bayesian inference. This is called active or embodied inference. Therefore, since embodiment complements and reinforces Bayesian inference, one can state that a stubbornly predictive coding is stably generated in the brain. These ideas are also implemented in robotics (Linson et al., 2018; Çatal et al., 2021).

While Bayesian inference seems to be flexible and to be far from rigorous logical thinking, it entails nothing but classical logic, or in other words, Boolean algebra, in which any phenomenon can be explained by a combination of atomic propositions taking the values yes or no and subject to the law of excluded middle (i.e., classical logical reductionism). Bayesian inference itself is not flexible in its logic, but it is a flexible *method* to determine within a predictive area what can be assessed and accepted by classical Boolean algebra (Arecchi, 2003, 2011; Gunji et al., 2017, 2020). In that sense, an image encoded inside the brain and an object existing outside of the brain must have a one-to-one correspondence through predictive coding.

In contrast, quantum cognition focuses on the other side of brain-function phenomena and/or cognition (Khrennikov, 2001, 2021; Aerts, 2009; Aerts et al., 2012, 2013, 2019; Haven and Khrennikov, 2013; Asano et al., 2015; Bruza et al., 2015). Quantum cognition describes and explains cognition, apprehension, comprehension, perception, and decision-making by using the basic formalism and conceptual logical and mathematical framework of quantum mechanics. It is not concerned with the physical basis of quantum processes in the brain's microscopic dynamics, and it does not apply quantum mechanics to macroscopic phenomena such as cognition and perception. This is why the quantum cognition community argues in favor of quantum mechanics being properly utilized only as a mathematical tool to model cognitive phenomena. Quantum logic, on which quantum cognition is based, fundamentally differs from Boolean logic (Boolean algebra). While Boolean logic has the structure of a rather simple complemented distributive lattice, quantum logic has the structure of a non-distributive lattice, i.e., a more complicated orthomodular lattice. This more complex structure implies that the cognition of multiple events entails a kind of resonance. This means that these multiple events interfere with each other due to the non-distributive nature of their logical evaluation, resulting in mutual or multi-interdependence. Resonance typically implies a non-linear interdependence (here in evaluating probabilities); distributive lattices have linear dependencies for their propositions and hence no resonance-type effects. The distributive law guarantees the independence of its reduced atomic events or, in other words, atomic propositions. In contrast, a non-distributive lattice guarantees the emergence of interactions among reduced atomic events, which entails resonance-like aspects for the probability of independent events. This can explain cognitive illusions such as the conjunction fallacy, in which the joint probability of the occurrence of events *A* and *B* is larger than that of the occurrence of *A*.

Quantum logic (i.e., an orthomodular lattice) results from a property of Hilbert space for the operators in quantum mechanics. While there is no fundamental physical reason to assume Hilbert space in a macroscopic universe, classical mechanics can still be formulated in a Hilbert space framework, as Koopman and Von Neumann proposed in the 1930s. However, this results in an operational probabilistic theory endowed with a classical Boolean algebra, which is complete as a lattice. The dependence on the Hilbert space framework for quantum cognition was considered a problem, or at least an inconvenience, in assigning meaning to the related operators and spaces. Quantum logic has been exemplified by Dirac's famous 3-polarizer experiment, but recently (Zhang, 2021a), an analysis for bipolar crisp and fuzzy sets has provided new insights into the old question. Indeed, as is well known, fuzzy set theory models vagueness by membership measures, while rough sets model incomplete information by bounding it with a lower and an upper approximation. Therefore, it would be very interesting to consider our previous work on rough set approximation and quantum cognition (Gunji and Haruna, 2010; Gunji et al., 2016; Gunji and Nakamura, 2022a,b) in this new light in future investigations. This could prove instrumental in taking further steps toward a deeper understanding of the interrelation of Bayesian inference and causal inference.

However, it has been recently verified that quantum logic, or orthomodular lattices, can be constructed without Hilbert space. First, this was achieved (Gunji et al., 2016) by the extension of Arecchi's idea of inverse Bayesian inference (Arecchi, 2003, 2011), and then it was achieved by the idea of ambiguity between what is inside and outside of a context (Gunji and Haruna, 2022; Gunji and Nakamura, 2022a,b). It can be achieved with respect to “rough set” lattices—a kind of special coarse-graining operation on regular sets—based on a binary relation. Quantum logic without Hilbert space has also been achieved by using category theory (Heunen and Vicary, 2019). This implies that there is now a clear and reasonable foundation by which quantum logic structures can readily be applied to macroscopic phenomena. Now, we can turn to the following questions that arise:

How are Bayesian inference and quantum cognition interrelated in macroscopic world phenomena?

While classic Bayesian inference leads to Boolean logic, in which classical logical reductionism holds, quantum logic can never be compatible with classical logical reductionism. Does this imply that the coexistence of Bayesian inference and quantum logic in a macroscopic setting entails an antinomy? How is Bayesian inference, which can be cast in a free-energy-principle form, interrelated with quantum logic or orthomodular lattices? If a datum is not related to the prior, then there is no context or its probability is recorded as zero. In other words, the probability of an event outside the context is almost zero. Thus, the underlying logic leads to a Boolean lattice. In contrast, quantum logic (or an orthomodular lattice) allows for contextuality since it accepts the non-zero probability of an event even outside the originally set context. We show here that such a non-zero probability of an event outside the original context can be obtained from an “*excessive Bayesian procedure”* or, in other words, an “*extended Bayesian inference”*. This entails a variation of the so-called “*Bayesian-Inverse-Bayesian”* non-linear loop (Gunji et al., 2016; Basios and Gunji, 2021). It might seem paradoxical, but upon closer examination, it is not. Although a one-to-one correspondence is enforced within the context, the non-zero probability of an event outside the given context can still be readily obtained. Stubborn predictive coding is resistant to change, and seemingly paradoxically, it not only affords but also actually gives rise to the possibility of considering other “outsider” events in addition to the events inside the context. This results in an instance of quantum logic.

This article is organized as follows: First, we show the relationship between the free energy minimization principles—Bayesian inference and Boolean algebra. Second, we implement an excess Bayesian procedure and demonstrate how the relationship between the datum and hypothesis is changed through this procedure. Third, we show that the excess Bayesian procedure entails an orthomodular lattice as a quasi-disjoint union of Boolean algebras. Therefore, in conclusion, we establish that this implies that quantum cognition and the free energy principle are connected to each other *via* an excess Bayesian procedure.

## Quantum cognition, orthomodular lattice (quantum logic), and free energy minimization

Quantum cognition, which is a new trend in cognitive science, is based on the notion of probability in quantum mechanics. Since any state of an event is defined as a vector of complex numbers, the probability of an event is expressed as the norm of the vector, as in quantum mechanics. Since the effect of quantum entanglement plays an essential role in calculating the joint probability, quantum cognition can explain various cognitive illusions. However, quantum cognition uses quantum mechanics not as a physical foundation of cognition but as information theory.

The orthomodular lattice is directly obtained from quantum mechanics; a lattice is an ordered set that is closed with respect to binary operations, *meet* and *join* (Appendix A). Given a complex number linear space, an element of an ordered set is defined by a set of vectors, and the order relation is defined by inclusion. For any set of vectors, a set of vectors that are orthogonal to the vectors is defined as an orthocomplement of them. The *meet* of sets of vectors is defined by their intersection, and the *join* of sets is defined by the composition of the orthocomplement of their union. Thus, an ordered set can be verified as an orthocomplemented lattice. Since a linear vector space is equipped with a Hilbert space, an orthocomplemented lattice is verified as an orthomodular lattice. If inclusion is regarded as a sequence of premises and consequences and *meet* and *join* are regarded as logical conjunction and disjunction, respectively, an orthomodular lattice is reformalized as quantum logic.

While quantum cognition and the orthomodular lattice were originally derived from quantum mechanics equipped with a Hilbert space, it has been verified that an orthomodular lattice can be obtained without a Hilbert space, and various cognitive illusions can be explained by subjective probability defined in an orthomodular lattice without a Hilbert space. This implies that quantum cognition may be established by an orthomodular lattice alone, without a Hilbert space.

Free energy minimization is a theory by which cognitive brain function is systematically explained, and it is based on Bayesian inference. The term free energy originates from the fact that the upper bound of the cost function that reveals the difficulty in predicting the sensory input is called variational free energy. First, we spell out how free energy minimization is related to Bayesian inference and how it is disconnected from quantum cognition. It is well known that the free energy minimization principle implies Kullback–Leibler divergence between the a priori probability and the a posteriori probability under the minimization of predictive error (Friston and Kiebel, 2009a,b). It is expressed as


(1) $$\begin{array}{l} min\left(KLD\left[p\left(u\right)∥p\left(u|s\right)\right]-\ln\;p\left(s\right)\right) \end{array}$$

Where, $KLD\left[p\left(x\right)∥q\left(x\right)\right]=\underset{i}{\sum }p\left(x_{i}\right)\log\frac{p\left(x_{i}\right)}{q\left(x_{i}\right)}$ is the Kullback–Leibler divergence between the two given probability distributions, *p, q*, and the random variable is *x*, while *s* is the given datum that has been experienced, and *lnp*(*s*) is the surprise resulting from a given experience (i.e., the predictive error), which is minimized. It is easy to see that *KLD*[*p*(*x*)∥*q*(*x*)] = 0 if, and only if, *p*(*x*) = *q*(*x*) almost everywhere.

Thus, the minimizing procedure (1) implies that the a priori probability coincides with the a posteriori probability. Here, we show that this procedure is nothing other than classic Bayesian inference. Since probability changes over time in Bayesian inference, let us introduce time as a suffix for the probability. The variables *d and h* represent the datum and hypothesis, respectively. The probability of datum *d* at time step *t* is represented by *P*^*t*^(*d*), and that of hypothesis *h* at time step *t* is represented by *P*^*t*^(*h* ).

The conditional probability *P*^*t*^(*h*|*d*) represents the probability of *h* under the experience of *d*. Since hypothesis *h* is the probability distribution of the data, hypothesis *h* is expressed in terms of the likelihood of data as *P*^*t*^(*d*|*h* ).

From the definition of the conditional probability of *A* given *B*, expressed as *P*(*A*|*B*), we have $P\left(d|h\right)=\frac{P\left(d,h\right)}{P\left(h\right)}$ and $P\left(h|d\right)=\frac{P\left(d,h\right)}{P\left(d\right)}$, so one obtains *via* Bayes' theorem that


(2) $$\begin{array}{l} P\left(h|d\right)P\left(d\right)=P\left(d|h\right)P\left(h\right) \end{array}$$

This is consistent with non-Bayesian probability theory when there is no iteration over time. However, because $P\left(d\right)=\underset{k}{\sum }P\left(d|h_{k}\right)P\left(h_{k}\right)$, and because we do have an iterative procedure over time in our case, with a time step *t*, we obtain for *P*^*t*^(*h*) the following expression:


(3) $$\begin{array}{l} P^{t}\left(h|d\right)=\frac{P^{t}\left(d|h\right)P^{t}\left(h\right)}{\underset{k}{\sum }P^{t}\left(d|h_{k}\right)P^{t}\left(h_{k}\right)} \end{array}$$

One might regard the calculation using Equation 3 as Bayesian inference, yet this is not a genuine Bayesian inference just because it is consistent with ordinary probability theory. The essence of Bayesian inference is that it allows us to compute, starting from a given a priori probability, a resulting a posteriori probability such that


(4) $$\begin{array}{l} P^{t+1}\left(h\right)=P^{t}\left(h|d\right) \end{array}$$

This is the goal of free energy minimization. The probability of the hypothesis *h* under a specific experience *d* is generalized as the probability of the hypothesis independent of experience.

The relation between data and hypotheses is analogous to the relationship between objects outside the brain and their representations (or “images”) inside the brain. Let us consider a set of hypotheses and data, *H* = {*h*_1_, *h*_2_, …, *h*_*N*_}, *D* = {*d*_1_, *d*_2_, …, *d*_*N*_}. A one-to-one correspondence between hypotheses and data is expressed by using the likelihood of a hypothesis and the probability of this hypothesis. The likelihood is expressed as


(5) $$\begin{array}{l} P^{t}\left(d_{i}|h_{j}\right)\sim 1.0,\left(i=j\right);P^{t}\left(d_{i}|h_{j}\right)\sim 0.0,\left(i\neq j\right). \end{array}$$

This one-to-one correspondence between hypotheses and data implies $P^{t}{\left(h\right)}_{j}=\;\frac{1}{N}$.

Therefore, the joint probability of a hypothesis is expressed as


(6) $$\begin{array}{l} P^{t}\left(d_{i},h_{j}\right)=P^{t}\left(d_{i}|h_{j}\right)P^{t}\left(h_{j}\right)\sim \frac{1}{N},\left(i=j\right);P^{t}\left(d_{i},h_{j}\right)\\ \;\sim 0.0,\left(i\neq j\right). \end{array}$$

**Figure 1:**
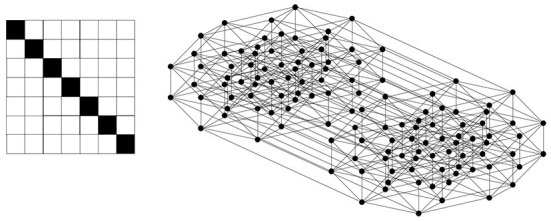
A diagonal relation and its corresponding lattice, which is a Boolean lattice. The lattice is shown here as a Hasse diagram, in which the elements of the lattice (a subset of *D*) are represented by black circles, and if one element is smaller than another element (i.e., one set is included in the other) and no element exists between them, then they are connected by a line, and the larger one is shown above the smaller one.

The joint probability between a hypothesis and data is transformed into a binary relation *R* ⊆ *H* × *D* such that (*h, d*) ∈ *R* if *P*^*t*^(*d, h*) > θ; otherwise, (*h, d*) ∉ *R*, . This results in a diagonal relation, as shown in Figure 1. This construction of a binary relation can be generalized to any joint probability between a hypothesis and data. In Figure 1, *H* = {*h*_1_, *h*_2_, …, *h*_7_}, *D* = {*d*_1_, *d*_2_, …, *d*_7_}, and from Equation 6, $P^{t}\left(d_{i},h_{i}\right)\sim 0.143;P^{t}\left(d_{i},h_{j}\right)\sim 0.0.$ Thus, given θ = 0.1, we obtain (*d*_*i*_, *h*_*i*_) ∈ *R* ⊆ *D* × *H* for *i* = 1, 2, …, 7 and (*d*_*i*_, *h*_*j*_) ∉ *R* for *i* ≠ *j*.

Now, given a binary relation between a hypothesis and data, one can estimate a logical structure with respect to a lattice (see Appendix A). There are several ways to construct a lattice from a binary relation, where a *concept* is defined as a fixed point with respect to certain defined operations. This has the result that a *concept* is expressed as a pair of a subset of the hypotheses, *H*, and a subset of the data, *D*. Since such a *concept* formulation for a rough set lattice is consistent with the “concept” discussed in cognitive linguistics, we apply the rough set lattice formalism and theory for a binary relation.

Given a set of hypotheses *H*, a set of data *D*, and a relation *R* ⊆ *H* × *D*, two operations, the upper and lower approximations (Appendix B) are defined as follows. For ∀*X* ⊆ *D*, the upper approximation of *X* with respect to a hypothesis is defined by


(7) $$\begin{array}{l} H^{*}\left(X\right)=\left\{T\in H|s\in X,sRT\right\} \end{array}$$

For ∀*Y* ⊆ *H*, the lower approximation of *Y* with respect to the data is defined by


(8) $$\begin{array}{l} D_{{\;}^{*}}\left(Y\right)=D-\left\{s\in D|T\in H-Y,sRT\right\} \end{array}$$

where *H*−*Y* represents the complement of *Y* in the set *H*.

A collection of fixed points of the composition of the two operations (7) and (8) is called a rough set lattice (Yao, 2004; Gunji and Haruna, 2010; see Appendix-B) and is described as


(9) $$\begin{array}{l} L=\left\{X\subseteq D|D_{{\;}^{*}}\left(H^{*}\left(X\right)\right)=X\right\} \end{array}$$

As shown in Appendix A, a lattice is defined by an ordered set that is closed with respect to specific binary operations, *meet* and *join*. In a rough set lattice, *L* is a subset of the power set of *D* and is closed with respect to *join* and *meet*. While there are other methods for constructing a lattice from a binary relation, such as the lattice of formal concept analysis that was developed to deal with cognitive memory (Kumar et al., 2015; Shivahare and Cherukuri, 2017), we used a rough set lattice for reasons based on cognitive linguistics.

In the ideal case of a diagonal relation, as shown in Figure 1, one obtains a Boolean lattice (Appendix A) of a rough-set lattice. First, $D_{*}\left(H^{*}\left(\left\{d_{i}\right\}\right)\right)=D*\left(\left\{h_{i}\right\}\right)=\{d_{i}\}$ for any *i* = 1, 2, …, 7. Thus, a singleton set of any element of *D* satisfies Equation 9 and is an element of a rough set lattice. Since a Boolean lattice is expressed as a power set of *D*, any subset of *D* is an element of *L*. This is easy to verify since for any subset of *D*, such as *X* = {*d*_*i*_, *d*_*j*_, …},


(10) $$\begin{array}{l} D_{{\;}^{*}}\left(H^{*}\left(X\right)\right)=D_{{\;}^{*}}\left(\left\{h_{i},h_{j},\ldots \right\}\right) \end{array}$$

and since for any *k* ∈ {1, 2, ..., *N*}, (*h*_*k*_, *d*_*k*_) ∈ *R*, and (*h*_*s*_, *d*_*k*_) ∉ *R*, (*s* ≠ *k*). Indeed, for any subset of *D*,


(11) $$\begin{array}{l} D_{{\;}^{*}}\left(\left\{h_{i},h_{j},\ldots \right\}\right)=D-\left\{s\in D|T\in \bar{\left\{h_{i},h_{j},\ldots \right\}},sRT\right\}\\ \;=D-\bar{\left\{d_{i},d_{j},\ldots \right\}}=\left\{d_{i},d_{j},\ldots \right\} \end{array}$$

where $\bar{\left\{d_{i},d_{j},\ldots \right\}}$ is the complement of {*d*_*i*_, *d*_*j*_, … }.

This implies that for any *X* ⊆ *D*,


(12) $$\begin{array}{l} D_{{\;}^{*}}\left(H^{{\;}^{*}}\left(X\right)\right)=X \end{array}$$

Therefore, *L* is the same as the power set of *D*, and *meet* is defined by intersection while *join* is defined by union. A Boolean lattice is a classical set-theoretic logic, and any *concept* within this logic, as defined above, can be expressed as a combination of logical atoms (i.e., the next least element of *D*), which in turn implies that any such *concept* can be reduced to atoms. This is why the Boolean lattice is simply classical logical reductionism. In the Hasse diagram of Figure 1, all elements of the rough set lattice defined by the power set of *D* = {*d*_1_, *d*_2_, …, *d*_7_} are represented by black circles. The least element is the empty set, and the elements, called atoms, just above the least element are {*d*_1_}, {*d*_2_}, …, {*d*_7_}. Then, the elements just above the atoms are all combinations of atoms, such as {*d*_1_, *d*_2_}, {*d*_1_, *d*_3_}, ..., {*d*_2_, *d*_3_}, …, {*d*_6_, *d*_7_}. The lattice contains all subsets of *D*, and the top element (i.e., the greatest element) is *D*.

A Boolean lattice is mathematically defined as a distributive complemented lattice. *Meet* and *join* constitute distributive laws for any element of the Boolean lattice. A *complemented* lattice implies that for any element there is at least one complement of it such that the *meet* of the element and its complement is the least element of the lattice, and their *join* is the greatest element of the lattice. In a Boolean lattice, for any element of the lattice, there is a unique complement called the *orthocomplement* (see Appendix A).

**Figure 2:**
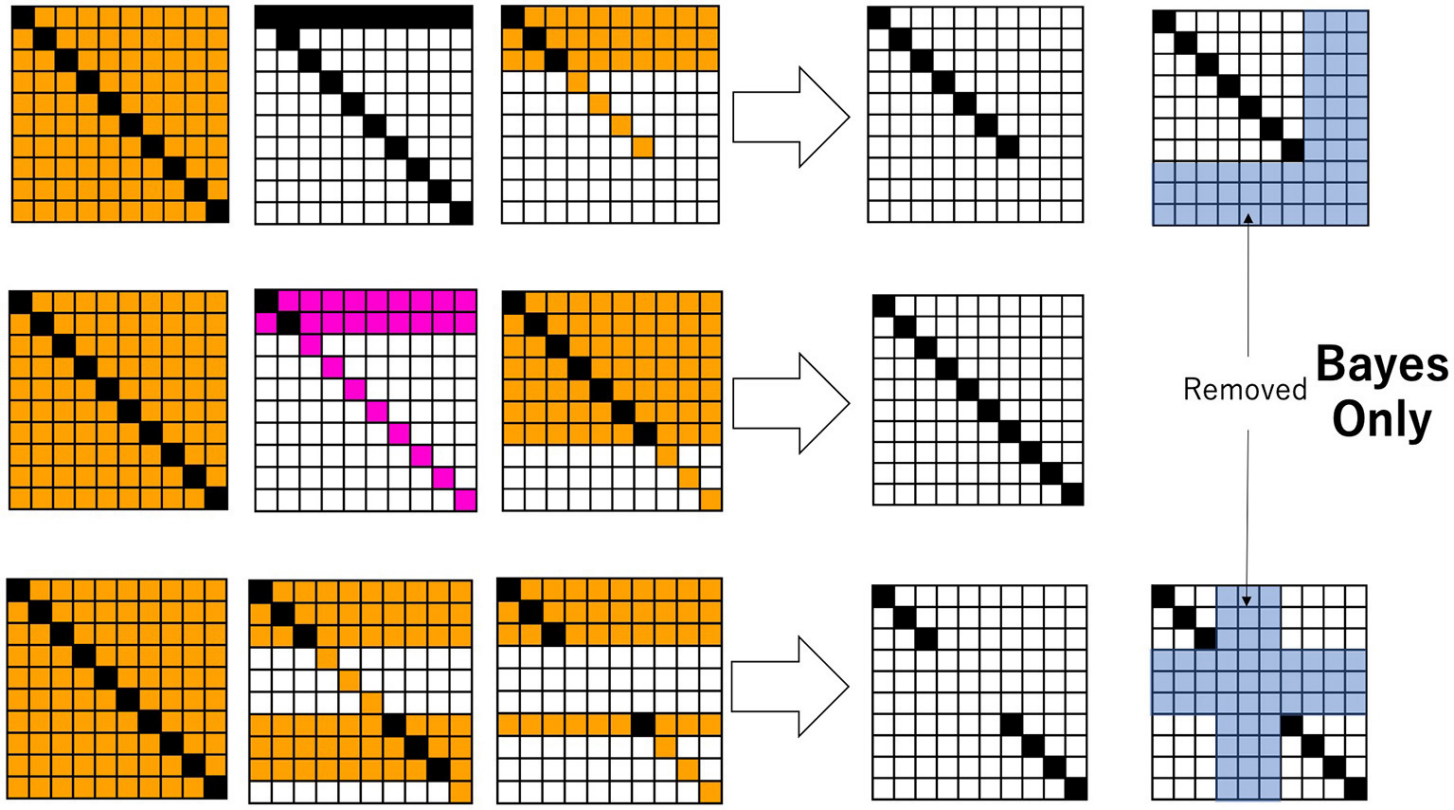
Snapshots of the joint probability between data and hypotheses. The probabilities are colored from low to high in the order of white, pale yellow, orange, pink, and black. By following the thick arrow, the binary relation *R* is obtained. The pale blue cells represent the domain in the relation, which is ignored by Bayesian inference.

Now, instead of an ideal diagonal relation, let us consider a binary relation between hypothesis and datum through Bayesian inference. Given an ideal diagonal relation as an initial condition, a specific sequence of data, *d* ∈ *D*_*E*_ ⊆ *D*, is given to obtain a decision-making system based on Bayesian inference. Figure 2 shows some snapshots, in the form of a heatmap, of the joint probabilities of hypotheses and data. In Figure 2, *H* = {*h*_1_, *h*_2_, …, *h*_10_}, *D* = {*d*_1_, *d*_2_, …, *d*_10_}, and initially, $P^{t=0}\left(d_{i}|h_{i}\right)=0.8$, and in the case of $i\neq j,\;P^{t=0}\left(d_{j}|h_{i}\right)=\frac{1-0.8}{9}=0.02$2. The probability of each hypothesis is such that *P*^*t* = 0^(*h*) = 0.1. The temporal development follows Equations 3, 4, where specific data, *d* in *P*^*t*^(*h*|*d*), are given at each time. In the top row in Figure 2, a specific *d* is randomly given from a subset of *D* such as *D* − {*d*_8_, *d*_9_, *d*_10_} = {*d*_1_, *d*_2_, …, *d*_7_}. Since the probability of *d* is calculated cumulatively, as time proceeds, the probability converges to the actual situation: $P^{t}\left(d_{8}\right)=\;P^{t}\left(d_{9}\right)=\;P^{t}\left(d_{10}\right)=$0.0, and $P^{t}\left(d_{s}\right)=$1/7=0.14 with *s* ≠ 8, 9, 10. If at time step *t*, *d*_*s*_with *s*≠8, 9, 10 is given, $P^{t}\left(h|d_{s}\right)=\frac{P^{t}\left(d_{s}|h\right)P^{t}\left(h\right)}{\underset{k}{\sum }P^{t}\left(d_{s}|h_{k}\right)P^{t}\left(h_{k}\right)}$ is calculated for any *h*. From this, $P^{t+1}\left(h\right)=P^{t}\left(h|d_{s}\right)$ is obtained by Equation 9. Finally, *P*^*t*+1^(*d, h*) is calculated by *P*^*t*+1^(*d*)*P*^*t*+1^(*h*). Each matrix of Figure 2 is obtained as a heatmap, in which if *P*^*t*+1^(*d, h*) ≥ 0.01, the cell is painted black; if 0.01 > *P*^*t*+1^(*d, h*) ≥ 0.008, it is painted pink; if 0.008>*P*^*t*+1^(*d, h*) ≥ 0.002, it is painted orange; if 0.002 > *P*^*t*+1^(*d, h*) ≥ 0.0006, it is painted pale yellow; and otherwise, it is painted white.

It is easy to see that data that have not been experienced or realized and their corresponding hypotheses are ignored through Bayesian inference. Therefore, a binary relation *R* is readily obtained for any *d*_*i*_ ∈ *D*_*E*_, (*h*_*j*_, *d*_*i*_) ∈ *R*, (*i* = *j*);(*h*_*j*_, *d*_*i*_) ∉ *R*, (*i* ≠ *j*), as shown in the right row of Figure 2. Henceforth, we call any such relation a sub-diagonal relation since a full diagonal relation holds only for a subset of *D*.

A rough set lattice corresponding to a sub-diagonal relation is also a Boolean lattice, the same as the lattice shown in Figure 1. It is easy to see that for any *X* ⊆ *D*_*E*_, $D_{*}\left(H^{*}\left(X\right)\right)=X$, and for any $Y\subseteq \bar{D_{E}}$, $D_{{\;}^{*}}\left(H^{*}\left(Y\right)\right)=D_{*}\left(\emptyset \right)=\emptyset$. Therefore, $D_{*}\left(H^{*}\left(X\cup Y\right)\right)\neq X\cup Y$, which implies that a rough-set lattice for a sub-diagonal relation on *D*_*E*_ is the power set of *D*_*E*_. Obviously, this is nothing more than a Boolean lattice by itself. The diagonal relation that entails a Boolean lattice also implies a one-to-one correspondence between objects and representations (or “images”), which is actually the basis for decision-making based on classical logical reductionism. When a decision-maker searches for an optimal solution through Bayesian inference, the domain in which the one-to-one correspondence holds is restricted to a small part of a whole binary relation. That small area results in a sub-diagonal relation, which helps the decision-makers avoid redundant searches. In other words, Bayesian inference is essentially the core of “stubborn empiricism.” While many researchers claim that predictive coding in the brain is flexible and plastic, with respect to search strategies in a changeable environment, Bayesian inference itself sticks to a “stubborn” optimization process.

## Excess Bayesian inference and quantum logic

Compared to Bayesian inference, or its equivalent free energy minimization, quantum cognition claims remarkably better flexibility and deviates from classical optimization in terms of decision-making processes. One of the most intriguing examples is the so-called “guppy effect,” which shows the essence of the conjunctive fallacy: given two events that are independent of each other, the joint probability of the two events occurring simultaneously is smaller than the probability of every single event (Aerts et al., 2012). The probability of an unknown person being male, represented by *P*(*male*), is $\frac{1}{2}$, and the probability that the person is born in March, *P*(*March*), is $\frac{1}{12}$. Therefore, since the probability that the person is male and is born in March is $P\left(male\wedge March\right)=\frac{1}{24}$, we have


(13) $$\begin{array}{l} P\left(male\wedge March\right)<P\left(male\right),P\left(male\wedge March\right)<P\left(March\right) \end{array}$$

The guppy effect contradicts this situation. If someone is asked to give an example of a fish, most likely, the person will respond with tuna, mackerel, or a similar example, and the probability that the person will give the example of a guppy, the popular pet fish that gave its name to this effect in the original publication, is very small. The probability of recalling the datum “guppy” when the prompt “fish” is given is represented by *P*_*guppy*_(*fish*). Analogously, if someone is asked to give an example of a pet, the person might respond with a cat or a dog, and probability of recalling *P*_*guppy*_(*pet*) is very small. However, if specifically asked to recall a pet fish, the person could recall the guppy with high probability, and this implies


(14) $$\begin{array}{l} P\left(pet\wedge fish\right)>P\left(fish\right),P\left(pet\wedge fish\right)>P\left(pet\right) \end{array}$$

This is the guppy effect.

In quantum cognition, quantum mechanics is used as the basis of information theory. An event is defined as a vector in Hilbert space, and the probability of an event is defined by the square of the norm of its vector. The guppy effect, in which a joint probability is larger than the probability of a single event, can be explained by the *entanglement* of events. Thus, we arrive at the realization that the probabilities of events are not independent of each other and are in fact an interaction among events. Their “*entanglement”* can occur through an effect outside the diagonal relation that results from classic Bayesian inference.

The difference between Bayesian inference and quantum cognition is clearly shown in terms of lattice theory. As mentioned previously, Bayesian inference entails a Boolean lattice. In quantum mechanics, one can define a set of vectors, *X*, as an element of a lattice, and an orthocomplement of *X*, represented by *X*^⊥^, is defined by the set of vectors whose inner product with any vector of *X* is zero (i.e., the two vectors are orthogonal to each other). *Meet*, again, is defined here by the intersection, and *join* is defined by the orthocomplement of the union. In Hilbert space, the resulting lattice is known to be an orthomodular lattice (Appendix A), which is essentially what quantum logic is. As mentioned previously, it has been recently demonstrated that quantum logic (an orthomodular lattice) can be constructed without a Hilbert space using category theory and/or lattice theory.

Since an orthomodular lattice is a non-distributive lattice, not all events and phenomena can be explained by a combination of logical atoms. In other words, the orthomodular lattice conflicts with the classical logical reductionistic approach supported by classical Boolean lattice theory. It can indeed accommodate flexible and plastic inference processes and interactions of thoughts concerning multiple events. In that sense, an orthomodular lattice or quantum logic not only conflicts with Boolean lattice theory but also surpasses it.

Thus far, we have obtained a lattice from a binary relation between data and hypotheses, and we have shown that Bayesian inference entails a Boolean lattice. Moreover, as has been clearly seen, Bayesian inference plays an essential role in human decision-making. Therefore, any other decision-making process that should be added to the Bayesian inference process must preserve and accommodate Bayesian inference itself. However, since decision-making based on quantum logic conflicts with decision-making based on Bayesian inference, these two decision-making processes might seem to be in conflict with each other. Therefore, the problem that arises is to determine what kind of process entailing quantum logic could be added to the basic Bayesian process.

Here, we define the *excess Bayesian process*, by which the tendency to stick to experience based on Bayesian inference is enhanced rather than canceled. The excess Bayesian process is defined by the detection of data that have been experienced and actually contracts the universe of discourse based on experience. Since the probability of a specific datum that is not observed decreases to almost 0.0 through Bayesian inference, only the joint probabilities of specific pairs of datums and hypotheses remain, which is called the effective domain. The excess Bayesian process regards the effective domain as a set consisting of all joint probabilities and modifies the likelihood function of the hypothesis so that it is contained in the effective domain. Therefore, the joint probability in the effective domain is divided by the summation of those joint probabilities in the effective domain. This is defined below, and the algorithmic representation is shown in Appendix C. While excess Bayesian inference has sometimes been referred to as inverse Bayesian inference (Gunji et al., 2016), inverse Bayesian inference is formalized so that it is symmetrical to Bayesian inference (Gunji et al., 2018, 2021). Since Bayesian inference uses a set of hypotheses to infer the environment by changing the probability of the hypotheses, the hypotheses are required to be sufficiently different from each other that each is sensitive to the environment. Therefore, each hypothesis *h*_*i*_ is required to have a sharp peak in the diagonal element datum *d*_*i*_. If the effective domain of the data is a subset of the set of all data, the likelihood of a hypothesis must be contained in the effective domain. This is simply an extension of Bayesian inference, and that is why it is called an excess Bayesian process. Therefore, we formulate the abovementioned excess Bayesian process as follows: Given a joint probability, $P^{t}\left(d_{i},h_{j}\right),i=1,2,\ldots ,N;j=1,2,\ldots ,N$, a binary relation is obtained by a given threshold value θ:


(15) $$\begin{array}{l} \left(h_{j},d_{i}\right)\in R^{t},ifP^{t}\left(d_{i},h_{j}\right)>\theta ;\left(h_{j},d_{i}\right)\notin R^{t},otherwise \end{array}$$

The detection of data is defined by taking a diagonal element (*h*_*i*_, *d*_*i*_) and considering inverting the relation either from $\left(h_{i},d_{i}\right)\in R^{t}$ to $\left(h_{i+1},d_{i+1}\right)\notin R^{t}$ or from $\left(h_{i},d_{i}\right)\notin R^{t}$ to $\left(h_{i+1},d_{i+1}\right)\in R^{t}$. This is expressed as a set **M**^*t*^ such that


(16) $$\begin{array}{l} {\text{M}}^{t}=\{M_{k}=\left(h_{i},d_{i}\right)|\left(\left(h_{i},d_{i}\right)\in R^{t}\wedge \left(h_{i+1},d_{i+1}\right)\notin R^{t}\right)\\ \;\vee \left(\left(h_{i},d_{i}\right)\notin R^{t}\wedge \left(h_{i+1},d_{i+1}\right)\in R^{t}\right)\} \end{array}$$

where if *M*_*k*_ = (*h*_*i*_, *d*_*i*_) and *M*_*k*+1_ = (*h*_*j*_, *d*_*j*_), then *i* < *j*. When the Bayesian inference is expressed as Equations 3, 4, some data are ignored, which decreases the probability of $P^{t}\left(d_{i},h_{j}\right)$ when *d*_*i*_ is not found under the given circumstances. A set **M**^*t*^ assigns a subset of the relation of diagonal elements. By constructing the set **M**^*t*^, we contract the universe of discourse depending on experience. In non-Bayesian probability theory, the following holds:


(17) $$\begin{array}{l} P\left(h,d\right)=P\left(h|d\right)\underset{k}{\sum }P\left(d,h_{k}\right) \end{array}$$

the following equation also holds:


(18) $$\begin{array}{l} P\left(d\right)=\underset{k}{\sum }P\left(d,h_{k}\right)=\underset{j}{\sum }P\left(d_{j}\right) \end{array}$$

The contraction of the universe depending on experience is implemented as follows: we assume that according to the belief that the universe consists only of experiences, the probability of the data that have been experienced is 1.0. That is,


(19) $$\begin{array}{l} P\left(d\right)=\underset{k}{\sum }P\left(d,h_{k}\right)=1.0 \end{array}$$

Substituting Equation 19 with Equation 17 and introducing an iterative process as before, with a given time step *t*, the newly contracted universe considering the data that have been experienced is denoted at each time step by *d*′, and one obtains


(20) $$\begin{array}{l} P^{t+1}\left(h,d\right)=P^{t}\left(h|d\right)=\frac{P^{t}\left(h,d\right)}{P^{t}\left(d^{{\;}^{\prime }}\right)} \end{array}$$

The probability of the contracted data, *P*^*t*^(*d*′), at each time step is calculated within the contracted universe of discourse by Equation 16, so we have


(21) $$\begin{array}{l} P^{t}\left(d^{{\;}^{\prime }}\right)=\sum_{j=p}^{q}P^{t}\left(h_{j},d\right) \end{array}$$

where the domain of summation of $P^{t}\left(h_{j},d\right)$ is given by


(22) $$\begin{array}{l} p={\pi M}_{k},q=\pi M_{k+1}with\;M_{k},M_{k+1}\in {\text{M}}^{t},\;k=1,\;2,\\ \;\ldots ,\;m-1 \end{array}$$

which assigns either the relation {$\left(h_{i},d_{i}\right)\in R^{t}$} that has been experienced or the relation {$\left(h_{i},d_{i}\right)\notin R^{t}$} that has not been experienced, where


(23) $$\begin{array}{l} \pi M_{k}=\pi \left(h_{i},d_{i}\right)=i \end{array}$$

which assigns the index of the diagonal element. For $M_{m}\in {\text{M}}^{t}$, *M*_*m*_ = (*h*_*N*_, *d*_*N*_), and then *p* = π*M*_*m*−1_, *q* = *N*. In the *excess Bayesian procedur*e, first, *P*^*t*^(*d*), as a part of the universe, is regarded as a universe, which entails *P*^*t*^(*d*) = 1.0, which cancels any effect of *P*^*t*^(*d*). Then, in turn, the data that have been experienced are denoted by *d*′, and then *P*^*t*^(*d*′) is calculated from the small area assigned by Equation 16. The following mnemonic expression might help us understand the basis of the excess Bayesian inference:


(24) $$\begin{array}{l} P\left(h,d\right)=\frac{P\left(h,d\right)}{P\left(d\right)}P\left(d\right) \end{array}$$

While Equation 24 is indeed trivial, *P*(*d*) in the numerator on the right-hand side must be regarded as 1.0, and *P*(*d*) in the denominator on the right-hand side must be regarded as *P*^*t*^(*d*′). Thus, we obtain Equation 20.

By using Equation 22 or 23, the joint probability is obtained by summation with respect to the hypotheses, so now it reads as:


(25) $$\begin{array}{l} P^{t+1}\left(h,d\right)=P^{t}\left(h|d\right)=\frac{P^{t}\left(h,d\right)}{P^{t}\left(d^{{\;}^{\prime }}\right)}=\frac{P^{t}\left(h,d\right)}{\overset{q}{\underset{j=p}{\sum }}P^{t}\left(h_{j},d\right)} \end{array}$$

Symmetrically, the joint probability is obtained by summation with respect to the data, so now it reads as:


(26) $$\begin{array}{l} P^{t+1}\left(h,d\right)=P^{t}\left(d|h\right)=\frac{P^{t}\left(h,d\right)}{P^{t}\left(h^{\prime }\right)}=\frac{P^{t}\left(h,d\right)}{\overset{q}{\underset{j=p}{\sum }}P^{t}\left(h,d_{j}\right)} \end{array}$$

Using Equations 25, 26, not only the joint probability in the assigned area but also the joint probability outside the assigned area is normalized. Therefore, the effect of Bayesian inference contributes to the area outside the originally assigned area. The algorithmic representation of excess Bayesian inference is shown in Appendix C.

**Figure 3:**
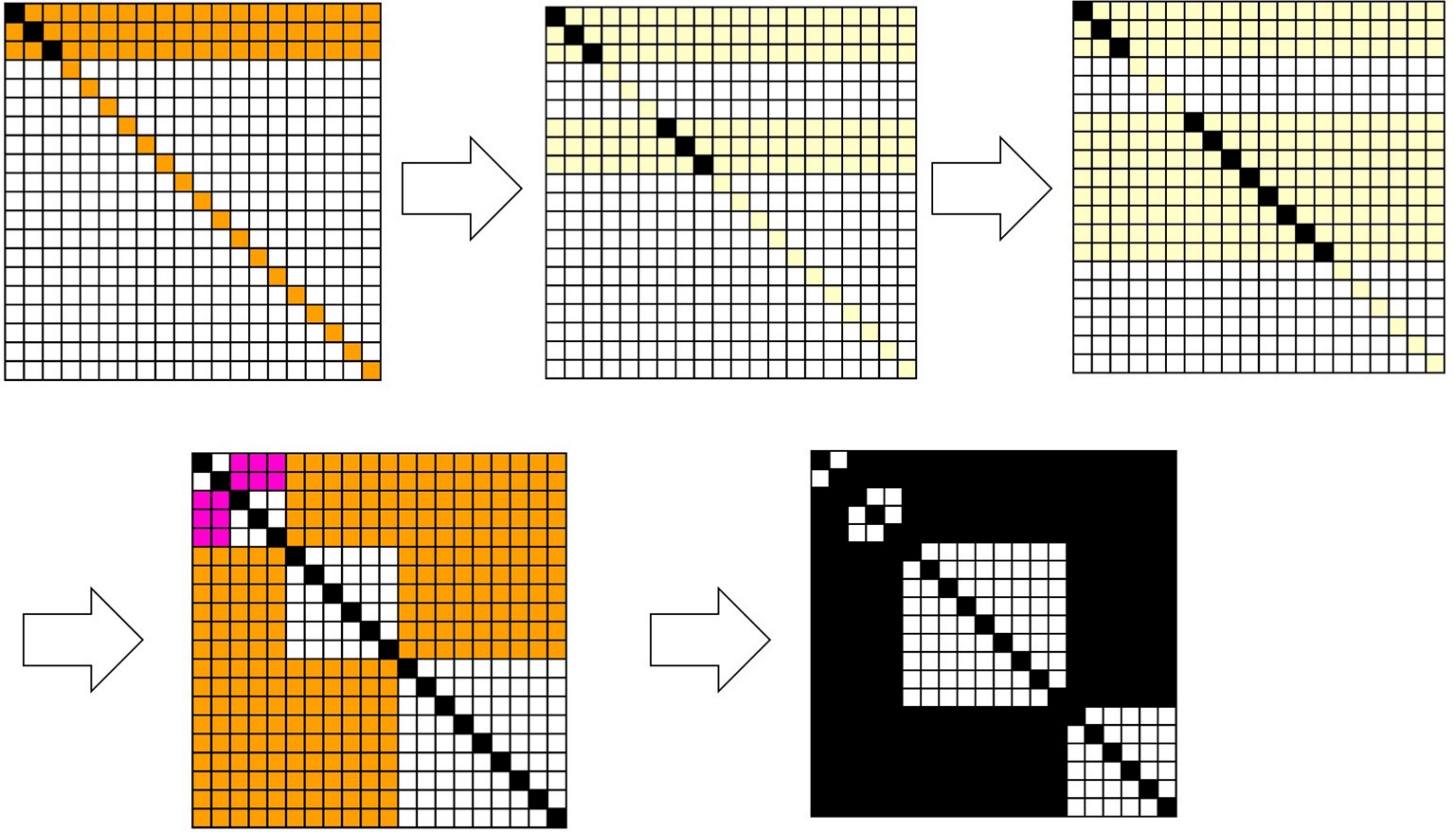
Snapshots of the joint probabilities of data and hypotheses and their corresponding relation *R*. The articulation of the data and hypotheses with respect to the diagonal elements is determined by Equation 22. The joint probabilities are colored from low to high in the order of white, pale yellow, orange, pink, and black. The last diagram in the sequence ordered by the arrows represents a binary relation. A black cell at (*h, d*) represents (*h, d*)∈*R*, and a white cell represents (*h, d*)∉*R*.

Figure 3 shows how Equations 25, 26 play a role in calculating the joint probability. This is a result of numerical simulation in which after the classic Bayesian process is used in the first 30 steps, the newly introduced excess Bayesian process, defined by Equations 25, 26, is implemented. In Figure 3, *H* = {*h*_1_, *h*_2_, …, *h*_20_}, *D* = {*d*_1_, *d*_2_, …, *d*_20_}, and initially, $P^{t=0}\left(d_{i}|h_{i}\right)=0.8$; in the case of $i\neq j,\;P^{t=0}\left(d_{j}|h_{i}\right)=\frac{1-0.8}{19}=0.011$. The probability of each hypothesis is such that *P*^*t* = 0^(*h*) = 0.05. The time development follows Equations 3, 4, where a specific data value, *d* in *P*^*t*^(*h*|*d*), is given at each time. In Figure 2, the specific *d* is randomly given from a subset of *D*, such as *D* − *E* with *E* = ({*d*_3_, *d*_4_, *d*_5_, *d*_6_} ∪ {*d*_15_, *d*_16_, *d*_17_, *d*_18_, *d*_19_, *d*_20_}). Since the probability of *d* is calculated cumulatively, as time proceeds, the probability converges to the situation; $P^{t}\left(d_{u}\right)=0.0$ for *d*_*u*_ ∈ *E* , and $P^{t}\left(d_{s}\right)=$1/10=0.10 for *d*_*s*_ ∉ *E*. However, Bayesian inference is adopted only for 30 time steps. If at time step *t*, *d*_*s*_∉*E* is given, then $P^{t}\left(h|d_{s}\right)=\frac{P^{t}\left(d_{s}|h\right)P^{t}\left(h\right)}{\underset{k}{\sum }P^{t}\left(d_{s}|h_{k}\right)P^{t}\left(h_{k}\right)}$ is calculated for any *h*. From this, $P^{t+1}\left(h\right)=P^{t}\left(h|d_{s}\right)$ is obtained by Equation 9. For the first 30 time steps, *P*^*t*+1^(*d, h*) is calculated by *P*^*t*+1^(*d*)*P*^*t*+1^(*h*). Each matrix in Figure 3 is obtained as a heatmap, in which if *P*^*t*+1^(*d, h*) ≥ 0.01, the cell is painted black; if 0.01 > *P*^*t*+1^(*d, h*) ≥ 0.008, it is painted pink; if 0.008 > *P*^*t*+1^(*d, h*) ≥ 0.002, it is painted orange; if 0.002 > *P*^*t*+1^(*d, h*) ≥ 0.0006, it is painted pale yellow; and otherwise, it is painted white.

In the excess Bayesian process, **M**^*t*^ is determined to assign data that have been experienced. When an element of ${\text{M}}^{t}=\left\{M_{1}=\left(h_{1},d_{1}\right),\;M_{2}=\left(h_{3},d_{3}\right),\;M_{3}=\left(h_{6},d_{6}\right),\;M_{4}=\left(h_{12},d_{12}\right)\right\}$ is chosen, one obtains *p* = π*M*_*k*_, *q* = π*M*_*k*+1_. As shown in Figure 3, a square whose vertices are (*h*_*p*_, *d*_*p*_), (*h*_*p*_, *d*_*q*−1_), (*h*_*q*−1_, *d*_*p*_), (*h*_*q*−1_, *d*_*q*−1_) becomes the diagonal relation that is defined by Equation 15. Squares are added due to this diagonal relation, such as the square whose vertices are {(*h*_1_, *d*_1_), (*h*_1_, *d*_2_), (*h*_2_, *d*_1_), (*h*_2_, *d*_2_)}, {(*h*_3_, *d*_3_), (*h*_3_, *d*_5_), (*h*_5_, *d*_3_), (*h*_5_, *d*_5_)}, {(*h*_6_, *d*_6_), (*h*_6_, *d*_11_), (*h*_11_, *d*_6_), (*h*_11_, *d*_11_)}, and {(*h*_12_, *d*_12_), (*h*_12_, *d*_20_), (*h*_20_, *d*_12_), (*h*_20_, *d*_20_)}, and consists of (*h*_*i*_, *d*_*j*_) ∈ *R*. The above example demonstrates how the joint probabilities of data and hypotheses in the area assigned by the data that have been experienced and their corresponding hypotheses are normalized by dividing those probabilities by $\overset{q}{\underset{j=p}{\sum }}P^{t}\left(h_{j},d\right)$ and $\overset{q}{\underset{j=p}{\sum }}P^{t}\left(h,d_{j}\right)$. Finally, each cell in the relation is painted black if *P*^*t*+1^(*d, h*) ≥ 0.0006; otherwise, it is painted white.

This normalization procedure enhances the diagonal relationship between the data and hypotheses beyond the experienced domain, which implies an explicit one-to-one correspondence between the data and hypotheses. In addition, the joint probabilities outside the diagonal relation are no longer negligibly small values. In Bayesian inference, only a diagonal relation is accessible as obtained, and any pair of data and hypotheses outside a diagonal relation are necessarily ignored; therefore, their joint probabilities almost disappear. In contrast, during the *excess Bayesian inference* process, pairs of data and hypotheses outside the diagonal relation are also enhanced, and the corresponding joint probabilities are increased. Although this might seem paradoxical and counterintuitive, it is indeed true that overestimating one's own experience allows alternative possibilities to emerge outside the given experience.

We previously showed that the psychological origin of quantum mechanics and/or quantum logic results not from a poor choice of basis vectors but from generating a context in which an object outside a brain is uniquely connected with a representation inside a brain, where outside the context, the object is connected to all images and the image is connected to all objects (Gunji and Haruna, 2022; Gunji and Nakamura, 2022a,b). In other words, there is a one-to-one relation within each context, and outside the context (i.e., background), objects are connected to all representations and vice versa. Since a one-to-one relation entails a Boolean algebra, this system entails multiple Boolean algebras connecting *via* the least and the greatest elements resulting from the background. This is the implementation of quantum logic without a Hilbert space. In this paper, we added another explanation for the psychological origin of quantum logic by introducing the excess Bayesian process.

The results from a numerical simulation of the excess Bayesian inference are shown in Figure 3, where the domain assigned by data that have been experienced is determined by Equation 22, which articulates the relationship between data that have been experienced and data that have not been experienced, and through this articulation, it assigns the vertex of the diagonal relation. Finally, the joint probabilities of the data and hypotheses entail, by induction, certain binary relations consisting of multiple diagonal relations and newly formed relations outside the diagonal relations, where pairs (*h*_*i*_, *d*_*j*_) outside the diagonal relations remain in their corresponding relations *R*.

**Figure 4:**
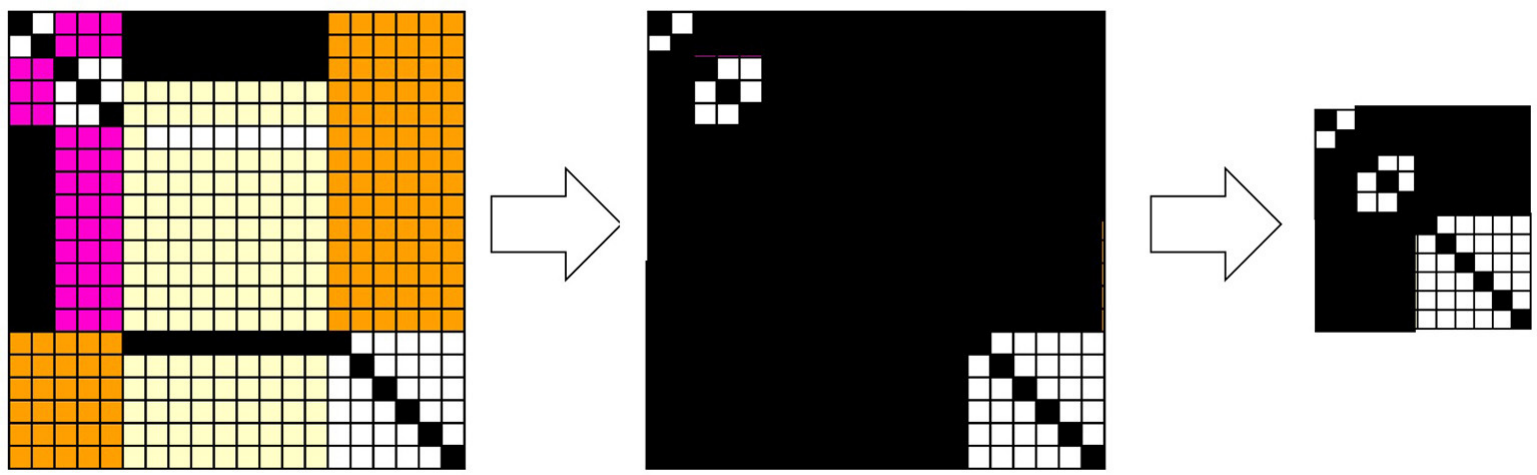
Snapshots of the joint probabilities of data and hypotheses and their corresponding relation *R*. The articulation of the data and hypotheses with respect to the diagonal elements is determined by Equation 23. The joint probabilities are colored from low to high in the order of white, pale yellow, orange, pink, and black (left). A black cell at (*h, d*) represents (*h, d*)∈*R*, and a white cell represents (*h, d*)∉*R* (center right).

Figure 4 shows the results from numerical simulations of the excess Bayesian inference, where the domain assigned by the experienced data is determined by Equations 22, 23, in which only data that have been experienced are assigned as articulated. Therefore, diagonal elements whose data have been experienced constitute a diagonal relation, while those whose data have not been experienced constitute the background outside the diagonal relations. Similar to Figure 3, any (*h*_*i*_, *d*_*j*_) outside the diagonal relations is in *R*. The central diagram in Figure 4 shows a large area consisting of cells representing (*h, d*) ∈ *R*, which contains diagonal cells. In this relation, for some *d*, any *h* is in a relation such that (*h, d*) ∈ *R*, and for some *h*, any *d* is in a relation such that (*h, d*) ∈ *R*. These pairs of (*h, d*) ∈ *R* can be canceled out with respect to a given rough-set lattice approximation because for some *d* such that any *h* has a relation to it, $D_{{\;}^{*}}\left(H^{*}\left(\left\{d,\;\ldots \right\}\right)\right)=D_{*}\left(H\right)=D$ is not an element of a lattice. Thus, a *d* such that any *h* has a relation to it and an *h* such that any *d* has a relation to it can be mutually removed from a relation *R*. The diagram on the right in Figure 4 shows such a relation, where the redundant rows and columns have been removed.

**Figure 5:**
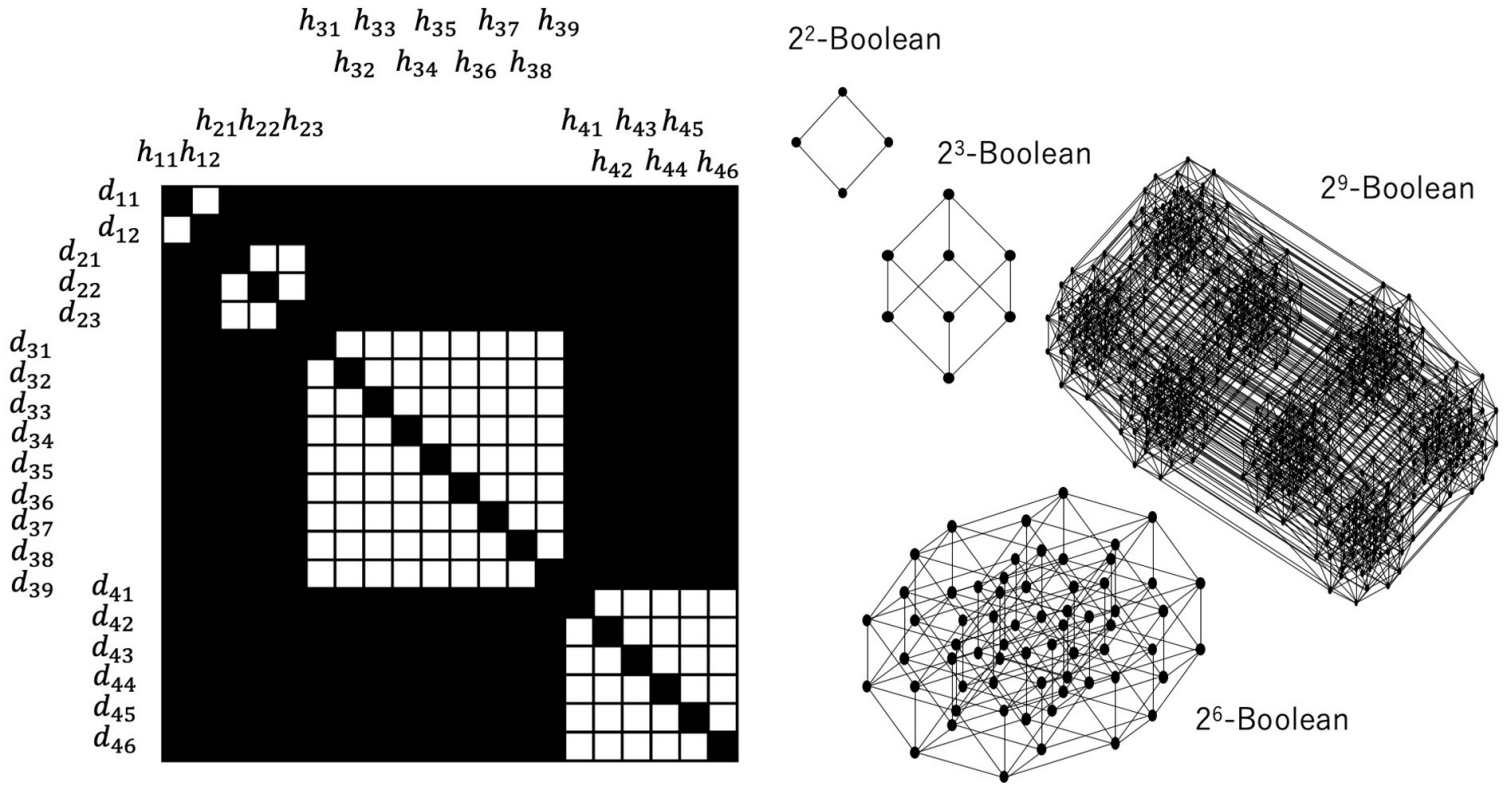
Relation between data and hypotheses consisting of (2 × 2), (3 × 3), (9 × 9), (6 × 6) diagonal relations and their corresponding Boolean lattices. All relations outside the diagonal relations constitute the greatest element, which fuses all the other Boolean lattices.

**Figure 6:**
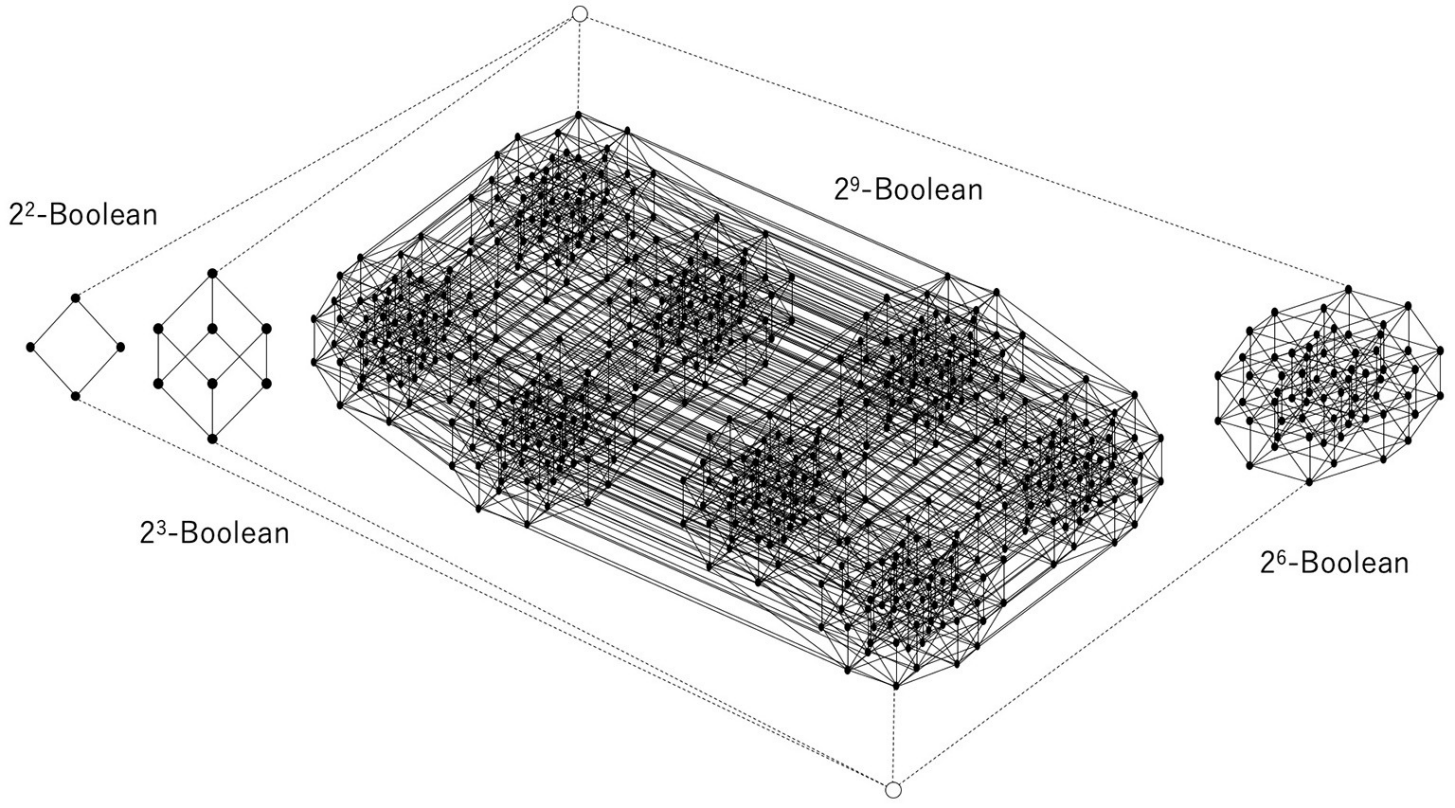
Disjoint union of some Boolean lattices, the least and greatest elements of which are common to all Boolean lattices. This is obtained from the relation shown in Figure 5.

Figures 5, 6 show a corresponding lattice obtained from the relation in Figure 3, where this lattice is defined by Equations 7–9. It is easy to see that they are the same kind of orthomodular lattices that correspond to quantum logic. Figure 5 shows a relation between the data and the hypotheses and their corresponding sublattices. When we focus on the 3 × 3 diagonal relation between {*d*_21_, *d*_22_, *d*_23_} and {*h*_21_, *h*_22_, *h*_23_}, the it is easy to see that


$$\begin{array}{l} D_{{\;}^{*}}\left(H^{*}\left(\left\{d_{21},d_{22}\right\}\right)\right)=D_{{\;}^{*}}\left(\left\{h_{21},h_{22}\right\}\cup \bar{\left\{h_{21},h_{22},h_{23}\right\}}\right)\\ =D-\left(\bar{\left\{d_{21},d_{22}\right\}}\cup \left\{d_{23}\right\}\right)=\left\{d_{21},d_{22}\right\} \end{array}$$

and that for a subset of *D, X*, consisting of elements belonging to different diagonal relations, Equation 12 does not hold, since $D_{*}\left(H^{*}\left(X\right)\right)=D$.

Let us also note here that $D_{*}\left(H^{*}\left(\left\{d_{21},d_{22},d_{23}\right\}\right)\right)=D$. Therefore, this 3 × 3 diagonal relation yields the power set of {*d*_21_, *d*_22_, *d*_23_} except for {*d*_21_, *d*_22_, *d*_23_}. Thus, if the greatest element is represented by *D*, the 3 × 3 diagonal relation entails a 2^3^-Boolean lattice. These considerations are general; therefore, an *n* × *n* diagonal relation entails a 2^*n*^-Boolean lattice.

Figure 6 shows the whole construction of a lattice corresponding to a whole relation. Each black circle represents an element of a (sub)lattice that is a subset of *D*. The greatest element and the least element are represented by white circles, and they are connected to the least and the greatest element of each Boolean lattice by broken lines, which implies that the greatest element of each Boolean lattice is the same as *D* and that the least element of each Boolean lattice is the empty set. Thus, one can say that the whole structure is a disjoint union of multiple Boolean (sub)lattices except for the least and the greatest element. It can be straightforwardly verified that this is an orthomodular lattice of the kind that is well known from quantum logic (see Appendix A).

Therefore, classical Bayesian inference gives rise to a Boolean lattice, while *excess Bayesian inference* gives rise to an orthomodular lattice. This is because classical Bayesian inference ignores the outside of the diagonal relation, which leads to a Boolean lattice. This implies that a decision-maker who applies a classical logical reductionistic approach to restricted pairs of data and hypotheses that are being experienced ignores the pairs of data and hypotheses that are not being experienced. In contrast, a decision-maker who not only uses classical Bayesian inference but augments it *via* an *excess Bayesian inference* process can enhance the basic one-to-one correspondence between the data and hypotheses by normalization within the domain of experience. This leads to multiple sub-relations, which are diagonal relations and relations that constitute the background of the diagonal relations. If the background consists of no relation [i.e., (*h, d*) ∉ *R*], multiple (sub)diagonal relations constitute the one diagonal relation expressing the Boolean algebra. However, if the background consists of relations [i.e., (*h, d*) ∈ *R*], this same background plays a key role in constituting the common greatest element by which multiple Boolean lattices are fused.

Since any element except for the least and greatest elements has a unique complement in each Boolean sublattice, any element can be regarded as an orthocomplement. Indeed, if an element and its orthocomplement in the lattice, such as those shown in Figure 6, can be compared with each other and with respect to the order, they are both in the same Boolean lattice participating as a sublattice, and then the distributive law holds for the element and its orthocomplement. This is why the orthomodular law holds for that lattice. Therefore, the result is that excess Bayesian inference can entail and accommodate quantum logic.

## Discussion and conclusion

We initially investigated the claim that the cognitive perspective based on the free energy principle could seemingly conflict with quantum cognition since the former tends toward optimization by removing redundant search space and the latter tends toward ambiguous and non-optimal decision-making. Since the free energy principle mathematically and formally includes classical Bayesian inference as an instance, one can estimate *via* optimization techniques the distribution of the joint probability of data and hypotheses, and this can be expressed as a binary relation. If the data and hypotheses are replaced by objects outside the brain and representations (or “images”) inside the brain, respectively, and if symmetry between objects and images is assumed, one can logically evaluate the hidden structure between objects and representations, “images,” with respect to a Boolean lattice structure. In this sense, one can estimate how a Boolean lattice (i.e., classical logic) and an orthomodular lattice (i.e., quantum logic) can arise from a given inference system. We also examined how these considerations can bridge the considerations of the free energy principle with those of quantum cognition. After a reviewer's comment (we thank them for this remark), it has come to our attention that bipolar fuzzy relations (Zhang, 1998, 2021a,b) should be considered and that comparing our previously proposed rough set approximation with respect to quantum cognition might enhance its scope by enabling a way to connect Bayesian inference and causal inference. Although this new development is beyond the scope of the present study, we maintain an interest in future investigations that could shed some light on the epistemological and ontological bases of quantum cognition and cognition at large.

Applying the free energy principle consists of minimizing the difference between the conditional probability under a given experience and the marginal probability *via* the minimization of the prediction error. Compared to cortical processes, in predictive coding, the former process is considered a classical Bayesian inference that has to be interpreted as a hierarchical top-down process, and the latter process is interpreted as a hierarchical bottom-up process. This is based on the premise that when new data are received and prediction errors are detected, the prediction errors lead to a modification of the prediction system. In other words, top-down Bayesian inference can make a system “see” new data through the old filters based on previous experiences. Thus, a system is subject to unavoidably becoming stuck in previous experience. Moreover, active inference (embodied cognition) is also a top-down process that enhances the Bayesian inference process. To receive data that are consistent with previous data, the top-down process makes the body move and act accordingly. This leads to stubborn inference based on experience. In this sense, an active inference might deploy a certain kind of possible excess Bayesian inference process. In other words, the active inference is a flexible interface based on a body between the environment and a stubborn inference system. This has been described as embodied intelligence.

In our work, we propose that excess Bayesian inference plays a key role in the process of cognition, much greater than that of active inference. This is because classical Bayesian inference restricts the domain of joint probabilities of data and hypotheses, while excess Bayesian inference realizes and enforces a one-to-one correspondence between data and hypotheses beyond the initially restricted domain. Therefore, this is called *excess* Bayesian inference. Our proposed process is implemented *via* a stepwise, iterative renormalization of the joint probability at each step divided by the sum of all joint probabilities in a restricted domain. This renormalization is achieved with respect to data and hypotheses independently, and these processes are not performed simultaneously. For any data, the joint probability is renormalized with respect to the hypotheses, and for any hypotheses, the joint probability is renormalized with respect to the data. Therefore, the effect of renormalization, which is inherent in the process of excess Bayesian inference, can influence not only the initially restricted domain but also the domains outside the initial domain. This implies that excess Bayesian inference can bring out non-zero joint probabilities that are noted as significant even outside the initially restricted domain, a potential that classical Bayesian inference lacks.

The distribution of the joint probabilities of data and hypotheses is expressed as a binary relation if a threshold value is introduced to distinguish a relation from the lack of a relation. Although Bayesian inference is expressed as a simple diagonal relation, excess Bayesian inference is expressed as multiple (sub)diagonal relations whose backgrounds consist of relations. The relations between the data and hypotheses are transformed into an algebraic structure called a lattice, and one can estimate the differences between classical Bayesian inference and the newly proposed *excess Bayesian inference* in terms of the lattice structure, each reflecting its underlying logical structure. Classical Bayesian inference is expressed as a simple Boolean lattice (classical logic), and *excess Bayesian inference* is expressed as an orthomodular lattice (quantum logic). This, in turn, implies that excess Bayesian inference can bridge the Boolean lattice structure of the classic Bayesian inference by encompassing a wider orthomodular lattice similar to those of quantum logic. From these considerations, we conclude that the basis of quantum cognition results from a radical extension of the Bayesian inference framework rather than simply alternative versions of classic Bayesian inference and that the free energy minimization principle can be bridged with quantum cognition *via* the proposed excess Bayesian inference scheme.

## Data availability statement

The original contributions presented in the study are included in the article/supplementary material, further inquiries can be directed to the corresponding author.

## Author contributions

Y-PG and SS constructed the idea of excess Bayesian inference and VB contributed on the development of ideas. Y-PG and VB wrote the manuscript. All authors contributed to the article and approved the submitted version.

## Funding

This work was supported by JSPS Topic-Setting Program to Advance Cutting-Edge Humanities and Social Sciences, JPJS00120351748. VB acknowledges partial support from the Italian CNR Foresight Institute's STEM materials program and from the Service de Physique des Systèmes Complexes et Mécanique Statistique of ULB.

## Conflict of interest

The authors declare that the research was conducted in the absence of any commercial or financial relationships that could be construed as a potential conflict of interest.

## Publisher's note

All claims expressed in this article are solely those of the authors and do not necessarily represent those of their affiliated organizations, or those of the publisher, the editors and the reviewers. Any product that may be evaluated in this article, or claim that may be made by its manufacturer, is not guaranteed or endorsed by the publisher.
