# Supplementary material for: Connecting the free energy principle with quantum cognition
Source: Front Neurorobot. 2022 Sep 2;16:910161. doi: 10.3389/fnbot.2022.910161 (PMC9478538; doi:10.3389/fnbot.2022.910161)
Supplement: Supplementary file 1 [file Presentation_1.pdf]

Appendix

Appendix A (Lattices)

(a) Ordered set and lattice

An ordered set P is defined as a set in which an order relation $\leq$ is given such that for any elements $a, b, c \in P$,

(i) $a \leq a$;

(ii) $a \leq b$ and $b \leq a$ implies $a = b$;

(iii) $a \leq b$ and $b \leq c$ implies $a \leq c$.

A lattice L is an ordered set in which, for any $x, y \in L$, "meet", $x \wedge y \in L$, and "join", $x \vee y \in L$ are defined, where $x \wedge y \leq x, x \wedge y \leq y$; if $z \leq x, z \leq y$, then $z \leq x \wedge y$ and $x \leq x \vee y, y \leq x \vee y$; if $x \leq z, y \leq z$, then $x \vee y \leq z$.

(b) Distributive lattice

A lattice L is a distributive lattice if and only if for any $a, b, c \in P$, $a \wedge (b \vee c) = (a \wedge b) \vee (a \wedge c)$.

(c) Complemented lattice

A lattice L is a complemented lattice if and only $\forall a \in L, \exists a^{\perp} \in L$ such that $a \wedge a^{\perp} = 0$ and $a \vee a^{\perp} = 1$, where 0 and 1 represent the least and the greatest element, respectively.

(d) Boolean lattice (classical logic)

A Boolean lattice is defined as a distributive complemented lattice.

(e) Orthocomplemented lattice

A lattice L is an orthocomplemented lattice if and only if $\forall a \in L, \exists a^{\perp} \in L$ such that

(i) $a \wedge a^{\perp} = 0$ or $a \vee a^{\perp} = 1$;

(ii) $a \leq b \Longrightarrow b^{\perp} \leq a^{\perp}$;

(iii) $a^{\perp\perp} = a$.

(f) Orthomodular lattice (quantum logic)

An orthocomplemented lattice L is an orthomodular lattice if and only if $a \leq b \Rightarrow b = a \vee (b \wedge a^{\perp})$, $\forall a, b \in L, a^{\perp} \in L$.

Appendix B (Rough set lattices)

(a) Equivalence relation

Given a set S, $R \subseteq S{\times}S$ is an equivalence relation if and only if for $a, b, c \in S$,

(i) aRa;

(ii) aRb implies bRa and vice versa;

(iii) aRb and bRc implies aRc.

(b) Equivalence class

Given an equivalence relation $R \subseteq S{\times}S$, an equivalence class of $x \in S$ with respect to R is defined by $[x]_R = \{y \in S | xRy\}$. A set viewed as an equivalence class is called a rough set.

(c) Approximation by a rough set

Given an equivalence relation $R \subseteq S{\times}S$, for any $X \subseteq S$, the lower approximation of X with respect to R, denoted by $R_*(X)$, is defined as $R_*(X) = \{x \in S | [x]_R \subseteq X\}$, and the upper approximation of X with respect to R, denoted by $R^*(X)$, is defined as $R^*(X) = \{x \in S | [x]_R \cap X \neq \emptyset\}$.

(d) Rough set lattice

Given two kinds of equivalence relations $R \subseteq S{\times}S$ and $K \subseteq S{\times}S$, $L = \{X \subseteq S | R^*\big(K_*(X)\big) = X\}$ can be verified to be a lattice and is called a rough set lattice. In a rough set lattice, any element is a set, and the order relation is defined by inclusion ($\subseteq$). Meet and join are defined as follows: For any $X, Y \subseteq S, X \wedge Y = R^*\big(K_*(X \cap Y)\big)$ and $X \vee Y = R^*\big(K_*(X \cup Y)\big)$.

Note: In the text of this paper, a relation R between a set of one equivalence class and a set of other equivalence classes is given, and the upper and lower approximations are replaced by H^* and D_*, respectively, for the sake of convenience.

Appendix C (Algorithmic representations for excess Bayesian inference)

//$H = \{h_1, h_2, \dots, h_N\}, D = \{d_1, d_2, \dots, d_N\}$
//$\boldsymbol{M}^t = \{M_1 = (h_1, d_1), M_2 = \big(h_p, d_p\big), \dots, M_m = (h_N, d_N),\}$
for ($k = 1$; $k \leq m$; $k++$) {

// excess Bayesian inference with respect to a datum
for ($j = 1$; $j \leq N$; $j++$) {
$sum = 0$;
for ($i = \pi M_k$; $i \leq \pi M_{k+1} - 1$; $i++$) {

$sum = sum + P(d_i, h_j)$;
}

for ($i = \pi M_k$; $i \leq \pi M_{k+1} - 1$; $i++$) {

$PP\big(d_i, h_j\big) = P\big(d_i, h_j\big)/sum$;
}
}

for ($i = \pi M_k; i \le \pi M_{k+1} - 1;\ i++$) {
for ($j = 1; j \le N;\ j++$) {
$P(d_i, h_j) = PP(d_i, h_j)$;
}
}
// excess Bayesian inference with respect to a hypothesis
for ($i = 1; i \le N;\ i++$) {
$sum = 0$;
for ($j = \pi M_k; j \le \pi M_{k+1} - 1;\ j++$) {
$sum = sum + P(d_i, h_j)$;
}
for ($j = \pi M_k; j \le \pi M_{k+1} - 1;\ j++$) {
$PP(d_i, h_j) = P(d_i, h_j)/sum$;
}
}
for ($j = \pi M_k; j \le \pi M_{k+1} - 1;\ j++$) {
for ($i = 1; i \le N;\ i++$) {
$P(d_i, h_j) = PP(d_i, h_j)$;
}
}
// Amplifying the effect
for ($i = \pi M_k; i \le \pi M_{k+1} - 1;\ i++$) {
for ($j = \pi M_k; j \le \pi M_{k+1} - 1;\ j++$) {
$P(d_i, h_j) = P(d_i, h_j) * P(d_i, h_j)$;
}
}
}
